# Current and novel polymeric biomaterials for neural tissue engineering

**DOI:** 10.1186/s12929-018-0491-8

**Published:** 2018-12-20

**Authors:** Rossana Boni, Azam Ali, Amin Shavandi, Andrew N. Clarkson

**Affiliations:** 1 Bioengineering Research Team, Centre for Bioengineering and Nanomedicine, Department of Food Science, University of Otago, PO Box 56, Dunedin, 9054 New Zealand; 2 Department of Anatomy, Brain Health Research Centre and Brain Research New Zealand, University of Otago, PO Box 56, Dunedin, 9054 New Zealand; 3 BioMatter-Biomass Transformation Lab (BTL), École interfacultaire de Bioingénieurs (EIB), École polytechnique de Bruxelles, Université Libre de Bruxelles, Avenue F.D. Roosevelt, 50 - CP 165/61, 1050 Brussels, Belgium

**Keywords:** Biomaterials, Synthetic and natural polymers, Neural tissue engineering, Axonal regeneration, Neuronal differentiation

## Abstract

The nervous system is a crucial component of the body and damages to this system, either by of injury or disease, can result in serious or potentially lethal consequences. Restoring the damaged nervous system is a great challenge due to the complex physiology system and limited regenerative capacity.

Polymers, either synthetic or natural in origin, have been extensively evaluated as a solution for restoring functions in damaged neural tissues. Polymers offer a wide range of versatility, in particular regarding shape and mechanical characteristics, and their biocompatibility is unmatched by other biomaterials, such as metals and ceramics. Several studies have shown that polymers can be shaped into suitable support structures, including nerve conduits, scaffolds, and electrospun matrices, capable of improving the regeneration of damaged neural tissues. In general, natural polymers offer the advantage of better biocompatibility and bioactivity, while synthetic or non-natural polymers have better mechanical properties and structural stability. Often, combinations of the two allow for the development of polymeric conduits able to mimic the native physiological environment of healthy neural tissues and, consequently, regulate cell behaviour and support the regeneration of injured nervous tissues.

Currently, most of neural tissue engineering applications are in pre-clinical study, in particular for use in the central nervous system, however collagen polymer conduits aimed at regeneration of peripheral nerves have already been successfully tested in clinical trials.

This review highlights different types of natural and synthetic polymers used in neural tissue engineering and their advantages and disadvantages for neural regeneration.

## Introduction

Tissue engineering combines principles and techniques of cell biology, material science, and engineering to fabricate tissue substitutes that mimic the structural and physiological nature of native tissue with the fundamental aim to regenerate the functional properties of an injured or diseased tissue [1].The regeneration and repair of both the central nervous system (CNS) and peripheral nervous system (PNS) remain crucial challenges in tissue engineering. The underlying reason is that both CNS and PNS have limited capacity for self-regeneration in mammals, and lasting functional deficits are common after disease and injury [2].

Impairments to the CNS can occur in a number of ways, such as trauma due to falls, car accidents, and assaults, which are the leading causes of long-term disability in both urban and rural population worldwide [3]. In addition, sport-related traumatic brain injuries contribute significantly in developed countries when compared to developing countries [3]. Neurodegenerative diseases, such as Alzheimer’s Disease, Parkinson’s Disease, Huntington Disease, prion disease, amyotrophic lateral sclerosis, and frontotemporal dementia, are a serious health problem resulting in discrete cell loss in specific brain regions [4]. Neurodegenerative diseases are insidious and progressive disorders and their incidence is on the rise as we have an aging population [5]. Stroke is also a leading cause of adult disability because of the brain’s limited capacity for repair [6–8], with most surviving the stroke, but living with a lasting impairment [9]. Finally, brain tumours are amongst the leading causes of death and are the second most common cancer found in children [10].

In the CNS, reactive astrocytes and the formation of an inhibitory glia scar largely prevent regeneration of the damaged tissues [11]. Most of the current research has been focused towards preventing further damage and on the stabilisation of the affected area, with limited research directed towards understanding reparative processes to enhance recovery of lost functions associated with injury to the CNS.

In addition, the PNS is also vulnerable to different kinds of traumatic injuries due to the extensive presence of nerves throughout the body [12]. The most common types of traumatic peripheral nerve injuries are penetrating injury, crush injury, traction injury, ischemia, laceration, compression, and thermal injury [13–15]. Trauma due to motor vehicle accidents, penetrating trauma related to violence, falls, and occupational accidents are the most common causes of traumatic injuries to the PNS [16]. Nerve injuries have devastating consequences on a patient’s quality of life, due to sensory and motor function defects which could be severe enough to paralyze the affected limb, combined with the development of excruciating neuropathic pain [17].

Currently, end-to-end neurorrhaphy is considered the clinical gold standard for the treatment of nerve gaps smaller than 1 cm and autologous nerve grafting is the common treatment for nerve damage exceeding 1 cm [18]. However, limited availability of nerve grafts, donor site morbidity, possible neuroma formation, and immunological responses are some of the critical issues limiting autologous nerve grafting as a therapeutic approach [12].

With the limitation of current therapeutic approaches for CNS and PNS injuries to be translated into the clinic, significant work has been directed towards developing novel neural tissue engineering strategies as potential treatments for tissue regeneration. Polymers, both synthetic and natural in origin, have shown consistent positive results in neural tissue engineering, including neurite outgrowth, differentiation of human neural stem cells, and nerve gap bridging [19–24]. New strategies aimed at the treatment of CNS and PNS injuries include polymeric scaffolds [25–29], hydrogels [23, 30–33], nanoparticles [34–37], and nerve conduits [20, 38–41]. The purpose of this review is to present an overview of the literature concerning polymeric applications, focusing particularly on the most recent discoveries, for neural tissue engineering and functional regeneration of nerve tissue.

## Neural tissue engineering

**Fig. 1:**
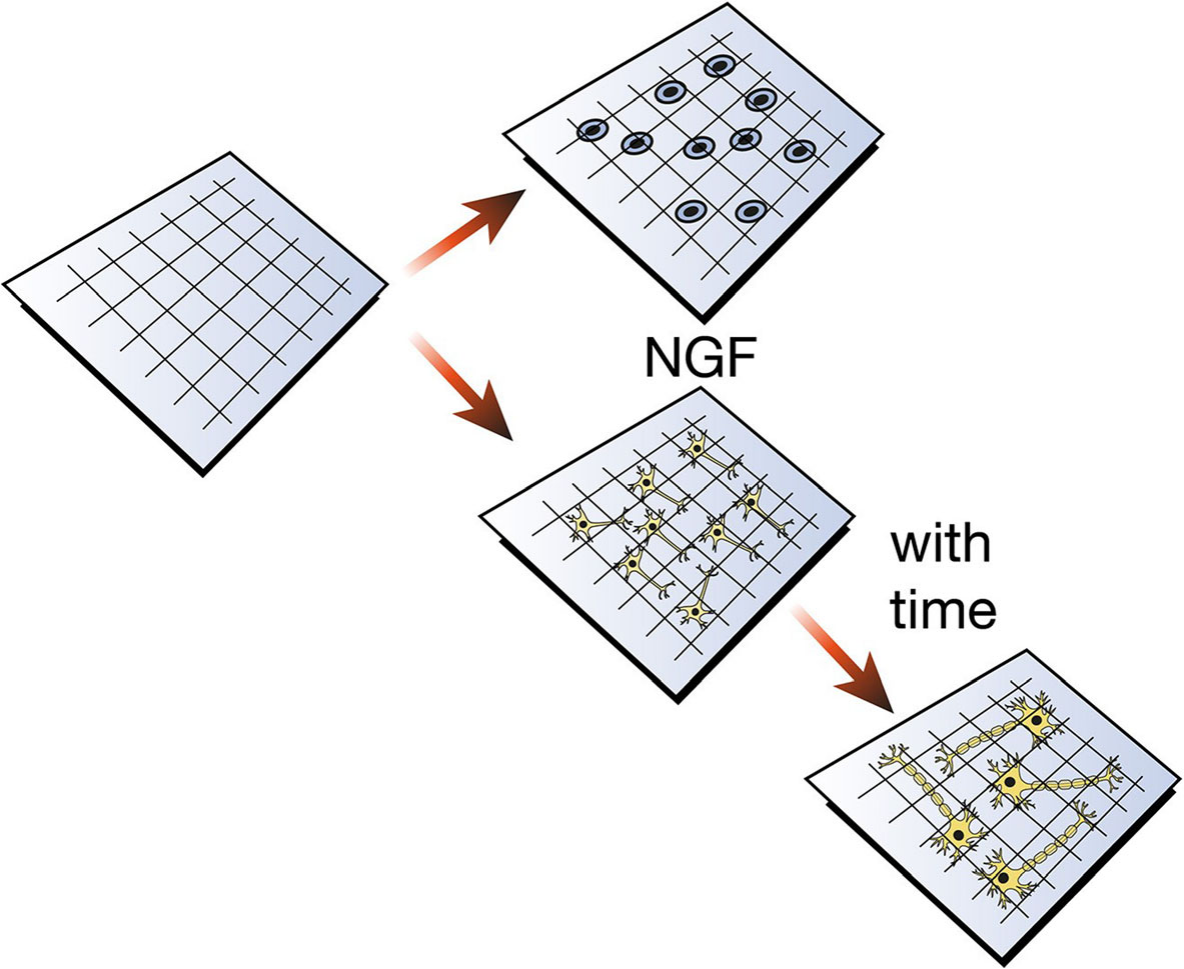
Polymeric structure for neural regeneration. Polymeric structures seeded with NGF offer mechanical support for growing neurites that in time will differentiate into fully matured neurons. They regulate biological cues to guide axonal growth and sprouting, to promote the regeneration of the nerve tissue

**Fig. 2:**
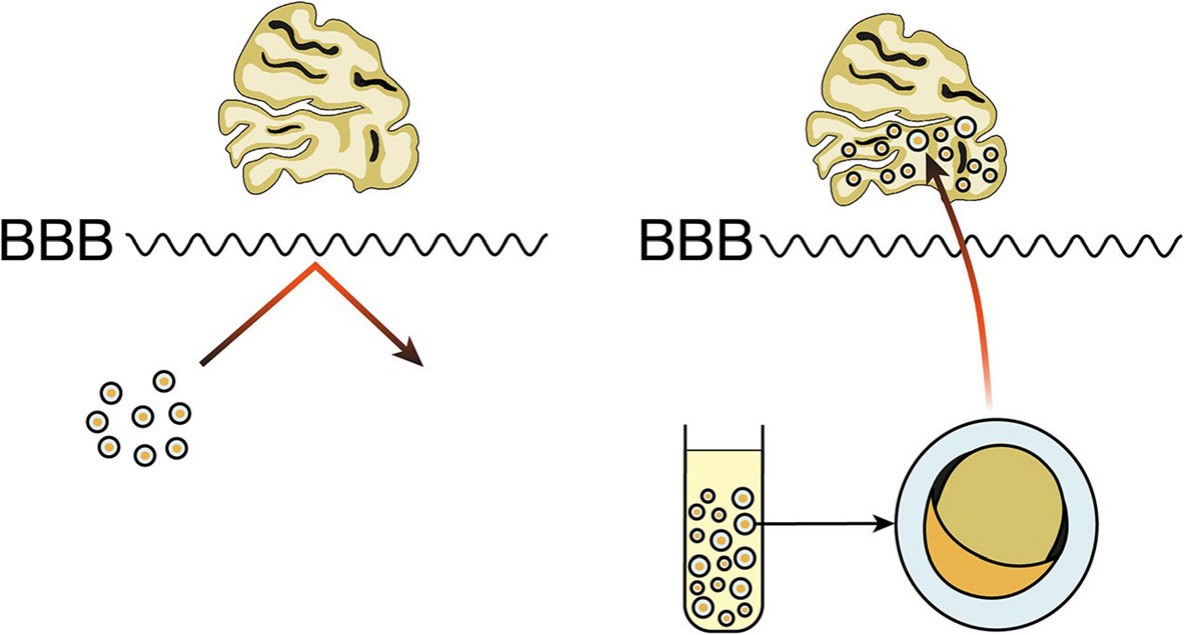
Polymer coating allows crossing of the BBB. Uncoated therapeutic drugs are unable to cross the BBB, but polymer nanoparticles are able to protect specific therapeutic agents, cross the BBB, and efficiently deliver drugs into damaged areas

During the last two decades, enormous progress has been made regarding our understanding of biological mechanisms regulating both CNS and PNS. Polymers have been largely used in neural tissue engineering due to their range of versatility that is unmatched by other biomaterials like metals and ceramics. The physical, chemical, mechanical, and inherent biological properties vary depending on the different polymer and each of these properties can be variated depending on the application they are being used for, such as 3D cell culture for PC12 cells, drug delivery vehicles, hydrogels, nerve conduits, and scaffolds. Successful polymeric structures not only offer mechanical support for growing neurites and inhibition of scar tissue, but they also regulate biological cues to guide axonal growth, promote regeneration, and stimulate integration into the existing healthy tissue [42, 43], Fig 1. Polymer nanoparticles are considered an optimal and versatile drug delivery system for regions like the brain. Polymer nanoparticles are able to protect therapeutic agents, cross the blood-brain barrier (BBB), and efficiently deliver drugs into damaged areas [44, 45], Fig. 2. Polymeric neural probes and electrodes have been successfully used as long-term chronically implantable neuroprosthetic devices for the treatment of neurodegenerative diseases, dystonia, chronic pain, and deep brain stimulation, becoming an invaluable clinical and diagnostic tool [46, 47].

## Natural polymers for neural tissue engineering

In neural tissue engineering, the use of natural polymers is highly beneficial due to their high biocompatibility and natural biodegradation kinetics combined with chemically tuneable properties. Often, natural polymers are analogues, if not identical like in the case of collagen, to substances already present in the human body, minimising the risks of cytotoxicity and immunogenic reaction upon implantation in the body [43]. In neural tissue engineering, natural polymers can fulfil different roles, including matrix formers, gelling agents, or drug release modifiers, and they can be easily adjusted to fit a defect in a difficult physiological geometry, such as the spinal cord [48, 49]. Natural polymers applied in neural tissue engineering have different origins, such as extracellular matrix components (ECM), like collagen, polymers derived from marine life, like alginate, polymers derived from crustaceans, like chitosan, and polymers derived from insects, like silk. Natural polymers are the most researched type of polymer in neural tissue engineering and they have been preclinically studied at length in numerous animal models, including primates. In addition, collagen is the only biopolymer currently approved for clinical studies aimed at peripheral nerve regeneration. However, weak mechanical characteristics due to complex chemical structures, thermal sensitivity, and processing difficulties that frequently require use of solvents, hinder the efficacy of natural polymers, prompting researchers to combine them with synthetic or electroconductive polymers. Table 1 summarises the main natural polymers used in neural tissue engineering and their applications.

**Table 1:** The main natural polymers used in neural tissue engineering, biocompatibility *in vitro/in vivo*, and examples of their applications

Natural Polymer	Biocompatibility *in vitro*	Biocompatibility *in vivo*	Application	References
Pre-clinical Studies				
Collagen		Non-human primates	Nerve guide	[51, 52]
		Rats	Hydrogel/scaffold	[23, 28]
		Dogs	Nerve conduits	[53]
		Cats	Nerve conduits	[54]
		Mice	Entubulation	[57]
	DRGs		Entubulation	[58]
		Rats	Entubulation	[59]
Fish Collagen	rBMSCs		No influence on neural differentiation	[63]
Gelatin	hC-MSCs		Electrospun conduits	[66]
	PC12		Electrospun conduits	[67]
	C17.2		Nerve conduits for Schwann cells	[68]
	RT4-D6P2T		Nerve conduits for Schwann cells	[168]
	PC12		Nerve conduits for Schwann cells	[177]
		Rats	Nerve conduit	[178, 181, 182]
	Allogeneic rMSCs		Scaffold	[27]
	Primary RSCs	Rats	Nanoparticles	[75]
	PC12		Hybrid Scaffold	[184]
	PC12		Bioink	[77, 78]
Elastin		Mice	Thermally Responsive ELPs	[193]
	PC12		ELPs Drug Depot	[194]
		Rats	ELPs Intranasal Drug Delivery	[195]
Hyaluronic Acid		Rats	Hydrogel	[197]
	NPCs from forebrain cortical neuroepithelium of E13.5 rats		Hydrogel	[198]
	C17.2 cells, ReNcells, GRPs		Hydrogel	[199]
	DRGs		Hydrogel	[39]
	NPCs		Hydrogel	[28, 202, 203]
		Rabbits	Nerve Conduit	[189, 206]
		Rats	Nerve Conduit	[48]
	RSC96		Nerve Conduit	[192]
	NSCs		Drug Delivery	[93, 94]
	PC12		Nanoparticles	[207]
	Cerebral cortices of Sprague–Dawley rats		Coating	[208]
Alginate		Cats	Gel	[209, 212, 213]
		Rats	Gel	[210]
		Rats	Sponge	[211, 215, 216]
	PDLMSCs and GMSCs		Hybrid scaffold	[217]
	PC12		Hybrid scaffold	[95]
	NSCs		Hybrid scaffold	[218]
		Rats	Hybrid scaffold	[108–111]
Chitosan	PC12		Hydrogel	[26, 76, 120]
	NPCs from forebrain cortical neuroepithelium of C57 fetal rats		Hydrogel	[113]
	NSCs		Hydrogel	[114, 115]
	Neuro-2a		Scaffold	[116]
	Schwann cells from Sprague-Dawley rats		Scaffold	[117, 118, 123–125].
	U373		Nanotubes	[119]
	BMSCs		Scaffold	[121]
		Dogs	Scaffold	[122]
	GFP+RG3.6		Micro/nano vehicle	[126]
		Rats	Micro/nano vehicle	[34, 127]
		Mice	Micro/nano vehicle	[128]
	hNSC		Bioink	[129]
Keratin		Mice	Nerve guide	[131, 132, 134]
	RT4-D6P2T	Mice	Hydrogel	[133]
		Rabbits	Hydrogel	[135]
		Primates	Hydrogel	[136]
	Glial cells		Nanofibrous Scaffold	[137]
Silk	NSCs		Hydrogel	[30, 141]
		Rats	Hydrogel	[39]
	DRGs		Hydrogel	[139, 140, 143, 145, 147]
	Primary Hippocampal Neurons		Scaffold	[144]
	PC12		Electrospun scaffold	[146]
		Rats	Electrospun scaffold	[39, 40]
		Dogs	Electrospun scaffold	[24]
		Rats	Nerve Guide	[148]
		Rats	Electrode Coating	[149]
	Glial Scarring		Electrode Coating	[150]
	NT2		Nerve guide (spider silk)	[151]
	NSCs		Matrix (spider silk)	[152]
Clinical Studies				
Collagen		Peripheral nerve reconstruction	NeuraGen®	[55]
		Peripheral nerve reconstruction	Neuromaix®	[56]

*DRGs*: dorsal root ganglia; *rBMSCs*: rat bone marrow mesenchymal stem cells; *hC-MSCs*: human chorion mesenchymal stem cells; *rMSCs*: rat mesenchymal stromal cells; *RSCs*: rat Schwann cells; *ELPs*: elastin-like polypeptides; *NPCs*: neural progenitor cells; *GRPs*: human glial-restricted precursors; *PDLMSCs*: human periodontal ligament mesenchymal stem cells; *GMSCs*: gingiva-derived mesenchymal stem cells; *NSCs*: neural stem cells; *BMSCs*: bone marrow stromal cells; *hNSCs*: human neural stem cells.

### Collagen

Collagen is a key structural biopolymer that makes up 30% of the mass of vertebrates, building their constitutional framework [50]. Humans have 28 proteins known as collagen and the most common is type I, a fibrillary type of collagen, the main component of connective tissues, which provide structure and support throughout the body, including bones, skin, tendons, cartilage, and nerves [50].

Collagen is a well-known biomaterial in neural tissue engineering. Early applications include the repair of a small (5mm) nerve gap in non-human primates through a collagen based nerve guide that proved to be physiologically similar to a graft repair [51, 52]. More recently, collagen conduits have been explored as a possible internal filler for neural conduits, increasing the quality of peripheral nerve regeneration over longer gaps. Collagen hydrogels improved the regeneration of a 15mm gap in rat sciatic nerve [23] and collagen conduits combined with NGF partially reconstructed a 35mm sciatic nerve defect in a dog model [53].Collagen is also used in combinations with other biopolymers and proteins. For instance, the electrophysiological evaluation of a collagen-PGA tube confirmed its role as a promising biomaterial for nerve conduits for peripheral nerve regeneration in cats [54] while a linear ordered collagen scaffold crosslinked with laminin, a key protein of the ECM in the nervous system, guided axonal growth and enhanced nerve regeneration as well as functional recovery in rats [28].

Collagen has been extensively studied as a biomaterial for neural tissue engineering and as a result, numerous collagen based nerve guides are commercially available on the market for peripheral nerves regeneration. Currently, collagen is the only biopolymer approved for clinical testing in neural tissue engineering. For example, NeuraGen® proved to be highly effective in peripheral nerve reconstruction in 43% of patients [55]. Another promising commercially available collagen nerve guide, Neuromaix®, showed outstanding results in bridging long nerve gaps in its first clinical trial [56]. It is clear that collagen based nerve conduits are the most biocompatible nerve conduit currently available in clinical settings, and its efficacy is often comparable to the clinical gold standard, autologous nerve grafting.

An interesting application of collagen is entubulation, hence the use of magnetically aligned type I collagen gel, achieved by exposing the forming collagen gel to a high-strength magnetic field, as a filler for collagen tubes. This method was successful in small peripheral nerve lesions, improving significantly nerve regeneration in a 6mm nerve gap in mice [57] and guiding neurite elongation and Schwann cell invasion *in vitro* [58] and *in vivo* [59].

Fish collagen has attracted interest as an alternative to its bovine counterpart. Fish collagen can be obtained from the by-products of fish and invertebrate processing, in form of skin, bone, and scales [60]. Fish collagen has been investigated as a potential biomaterial due to its advantageous biological characteristics, such as excellent biocompatibility, low antigenicity, high level of cell adhesion, and excellent biodegradability [61]. Fish collagen scaffolds, 2D or 3D, exhibit considerable cell viability, comparable to that of bovine collagen and they have been used for both soft and hard tissue applications [61, 62]. However, there is little to no work done on fish collagen for neural tissue engineering and, despite its promising features as a biomaterial, research carried out by Liu et al. stated that hydrolysed fish collagen promotes osteogenic and endothelial differentiation from bone marrow stem cells, but it does not function as a neural-inducing factor [63]. For the moment, the findings of Liu et al. combined with the relative lack of information regarding the use of fish collagen in neural tissue engineering limit its application in this field.

### Gelatin

Gelatin is a denatured protein obtained by hydrolysis of animal collagen with either acid or alkaline. Gelatin has a long history of safe use in pharmaceuticals, cosmetics, and food products due to its broad array of advantages, including low cost, availability, high biocompatibility, and biodegradability. Further, as a denatured product, gelatin is less antigenic than collagen and its chemically modifiable structure allows modulation of cell adhesion and proliferation, improving the biological behaviour of a polymeric device upon implantation [64].

**Fig. 3:**
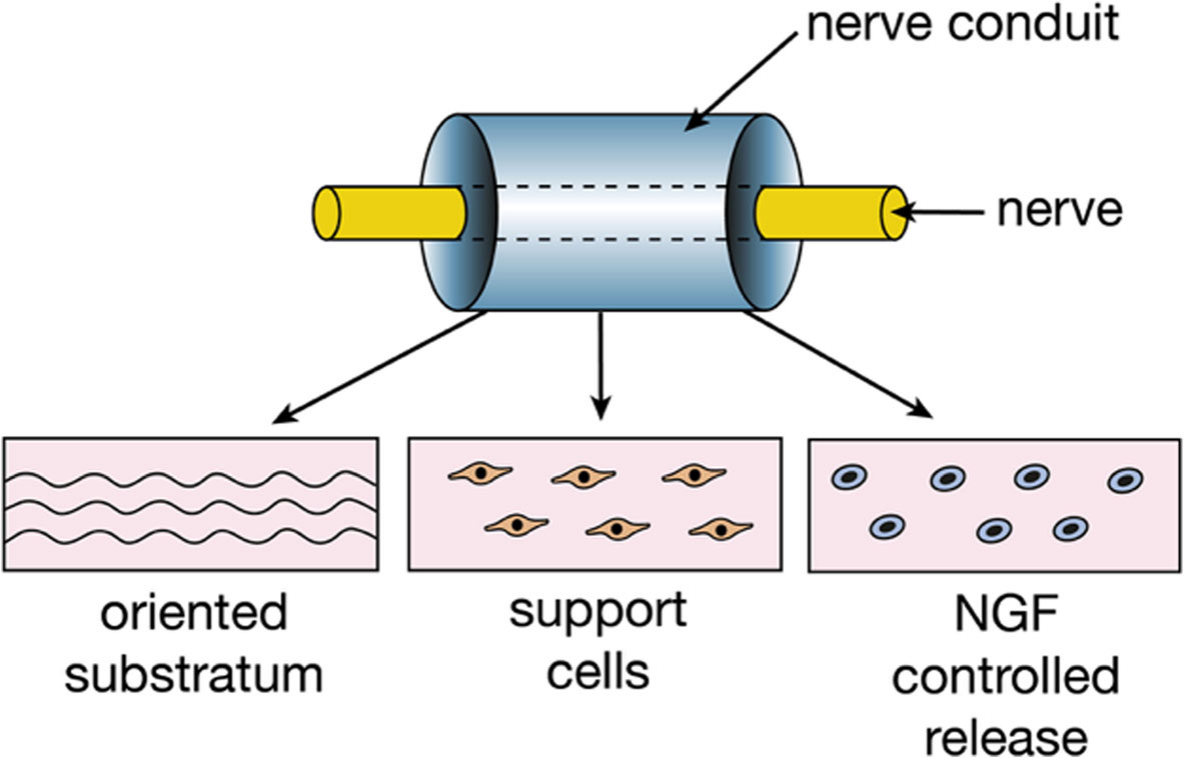
Polymeric nerve conduit. Components of a polymeric nerve conduit, oriented substratum, support cells, and controlled release of a neural growth factor

Primarily, gelatin has found applications in neural tissue engineering as electrospun combinations with other polymers, synthetic or natural in origin. The use of electrospinning as a fabrication technique for gelatin-based nerve conduits is particularly advantageous because it allows the optimisation and manipulation of mechanical, biological, and kinetic properties. In particular, electrospinning allows control over the orientation of the nanofibres, which is a key component in the creation of a functional scaffold [65]. Fig. 3 offers a simple overview of the structure of as nerve conduit focusing in its three crucial parts: oriented substratum achieved through electrospinning, seeded support cells, and controlled release of NGF. 3D electrospun nanofibrous gelatin conduits allowed differentiation of motor neuron-like cells, showing great potential for applications in the CNS [66, 67]. The most common combination of hybrid polymer conduits is gelatin/PCL. Gelatin combined with PCL acted as a positive cue to support neurite outgrowth and allowed culture and proliferation of Schwann cells *in vitro* [68–70] and, more recently, a PCL/collagen blend incorporated into a gelatin matrix was used to bridge a 15mm gap in sciatic nerve in rats [71]. Gelatin has also been successfully blended and electrospun with PLA, increasing differentiation into motor neurons lineages and promoting neurite outgrowth [72].

Gelatin is often crosslinked with genipin, a non-toxic crosslinker for proteins which enhances both biocompatibility and stability of the crosslinked product. An interesting application involved electrospun gelatin scaffolds crosslinked with genipin as a platform to provide biochemical cues to seeded cells in a decellularised rat brain ECM. This novel approach showed biocompatibility, cytocompatibility, and differentiative potential, providing tissue-specific signals aimed at expressing neural precursor cells [27]. Yang et al. developed a biodegradable nerve guide conduit containing gelatin crosslinked with genipin and tri-calcium phosphate ceramic particles for peripheral nerve regeneration. The conduit was tested on a short gap, 10 mm, in rat sciatic nerve, but it showed increased motor functionality and histomorphometric assessments confirmed its superiority over silicone tubes [73, 74].

Recently, gelatin nanoparticles have been used to enhance the biocompatibility of polymeric scaffolds for neural tissue engineering. For example, gelatin coated nanoparticles contained in cellulose acetate/PLA scaffolds showcased higher cell viability than uncoated scaffolds and they acted as a nerve guidance conduit for sciatic nerve defects *in vitro* and *in vivo* [75] while a gelatin/chitosan/PEDOT hybrid scaffold enhanced neurite growth of PC12 cells and promoted neuron-like cell adhesion and proliferation [76].

In addition, gelatin hydrogels have been used as a printable bioink for advanced bioprinting. Zhu et al. combined a gelatin/methacrylamide hydrogel with graphene nanoplatelets as a novel bioink and the printed neural constructs exhibited well-defined architecture, homogenous cell distribution, and neuronal differentiation [77]. The same research group combined this technology with low level light therapy which exhibited positive effects on the rehabilitation of degenerative nerves and neural disorders [78].

### Elastin

Elastin-based biomaterials are attracting a lot of interest for tissue engineering applications due to their remarkable properties. Elastin is a structural protein characterised by elasticity, self-assembly, long-term stability, and biological activity. Elastin is an ECM protein that provides elasticity to tissues and organs, therefore it is most abundant in organs where elasticity is a key aspect, such as blood vessels, elastic ligaments, lungs, and skin [79]. Clearly, incorporation of elastin in biomaterials is majorly significant when the elasticity effects can be exploited, hence its most popular applications are for soft tissue regeneration, such as skin and blood vessels [80, 81].

However, elastin-like polypeptides (ELPs) have found specialised applications in neural tissue engineering. ELPs enhance the biocompatibility and stability of polymeric structures and, due to their tuneable characteristics, act as robust drug delivery systems targeting the brain. For example, ELPs can be tailor made to be thermally responsive and passively target specific areas of the CNS for treatment of neurodegenerative disorders [82]. ELPs fused with neurotrophin served as a drug depot, limiting neurotrophin loss due to diffusion, and allowed controlled spatio-temporal drug delivery [83]. Moreover, the tuneable characteristics of ELPs allowed intranasal administration aimed at therapeutic delivery of drugs to the CNS [84].

Elastin is not widely used in neural tissue engineering, but ELPs have been recently investigated for novel drug delivery systems and they have found promising applications for thermal inhibition of neurodegenerative disorders. Therefore, it is conceivable that elastin has found its niche role in neural tissue engineering and its applications could expand to include different devices and regeneration strategies.

### Hyaluronic acid

Hyaluronic acid (HA) is a glycosaminoglycan found in extracellular tissues in various parts of the human body, where it plays a crucial role in lubrication. HA has been investigated at length for tissue engineering purposes due to its tuneable properties including biodegradability, biocompatibility, bioresorbability, and hydrogel forming ability [85].

HA has found widespread success in neural tissue engineering, supporting neurite outgrowth, differentiation, and proliferation on different substrates. HA hydrogels enhance the survival rates and proliferation of neural precursors, holding great promise for peripheral nerve regeneration therapies [86, 87] and therapeutic approaches to the CNS [32, 88, 89] In particular, HA hydrogels have suitable mechanical properties that influence the differentiation of neural progenitors, opening a new path for therapies targeting neurodegenerative diseases [21, 90].

HA can be combined with other natural biopolymers, especially collagen due to the similar nature of the two biomaterials. For instance, Zhang et al. used neural stem cells embedded in a HA/collagen conduit to promote the regeneration of a 5mm facial nerve gap in rabbits [91]. Combinations of HA and chitosan have also been successful in peripheral nerve regeneration. Li et al treated peripheral nerve crush injury in a rat model using chitosan conduits combined with HA [41], and Xu et al. used an injectable chitosan/HA biodegradable hydrogel for the regeneration of peripheral nerve injury [92]. Further, blends of HA and biodegradable synthetic polymers, such as PLGA and poly-L-lysine, showed great potential for controlled delivery of drugs aimed at axonal regrowth after spinal cord injury *in vitro* [93] and *in vivo* [94].

The high biocompatibility of HA has been invaluable to decrease the inflammatory response generated by electroconductive polymers in neural tissue engineering. For example, HA nanoparticles doped with PEDOT have been incorporated into a chitosan/gelatin scaffold, showing great PC12 cell adhesion and growth [95], and pyrrole/HA conjugates demonstrated considerable potential to mask conducting electrodes from adverse glial response upon implantation [96].

### Alginate

Alginate is a naturally occurring anionic biopolymer usually obtained from brown seaweed. Alginate has found growing interest in tissue engineering due to its biocompatibility, low toxicity, low-cost, and gelation characteristics [97]. However, one of the key disadvantages of alginate is the natural presence of impurities, such as heavy metals, endotoxins, proteins, and polyphenolic compounds, attributable to its marine origin. Therefore, alginate has to be purified in a multi-step extraction procedure to a very high purity in order to minimise possible adverse effects, including immunogenic or inflammatory responses, upon implantation [97].

Alginate has been used in various biomedical applications, such as drug and protein delivery, wound healing, and as a substrate for cell culture. Alginate gels were also found to be particularly useful for tissue engineering, promoting the regeneration of blood vessels, bones, cartilage, muscle, pancreas, liver, and peripheral nerves.

Suzukia et al., a Japanese research group, investigated at length the use of alginate in neural tissue engineering. Their studies showed that alginate gels promote peripheral nerve regeneration across a long gap, 50mm, in cat sciatic nerve [98] and a 10mm nerve gap in rats, increasing the diameter of the regenerating axons [99]. The group also tested an alginate sponge for the repair of facial nerves in cats. The facial nerve repaired with alginate showed remarkable regeneration but the group notably reduced the size of the nerve defect to 5mm [100]. Further, alginate sponges implemented to regenerate cavernous nerves in rats showed exceptional regeneration and restoration of erectile function [101], and they also successfully enhanced elongation of regenerating axons in the spinal cord of young rats [102], albeit both researches considered a very small gap (2mm).

Suzukia et al. also showed how non-tubulation repair using alginate has no significant differences for peripheral nerve injury regeneration compared to its tubulation counterpart on a cat model and it can be used as a regenerative approach [103]. These findings were confirmed by Hashimoto et al. who showed no electrophysiological or morphological differences between alginate tubular and non-tubular structures used for nerve regeneration [104].

Alginate has been recently used to create scaffolds for neural applications. Usually, hybrid scaffolds combine the biological characteristics of alginate with the mechanical properties of other biopolymers, both natural and synthetic in origin such as HA or PVA, showing great potential for peripheral nerve regeneration [105–107]. The leading application of alginate in neural tissue engineering is the treatment of spinal cord injury in rats, where it has been continuously successful in regenerating small nerve gaps, ranging from 2 to 4 mm [108–111].

### Chitosan

Chitosan is a linear polysaccharide derived by the chemical deacetylation of chitin, the major structural polysaccharide found in crustaceans and shellfish. Chitosan has very interesting properties, such as gel forming capabilities, high adsorption capacity, and biodegradability. Chitosan is extremely biocompatible and non-cytotoxic, as well as presenting antibacterial, antifungal, and antitumor activity [112]. In addition, chitosan is a versatile biopolymer easily processed into sponges, gels, membranes, beads, and scaffolds, therefore it can be tailor made to suit a specific application.

Chitosan hydrogels have been consistently successful in neural tissue engineering, exhibiting cell adhesion, cell interaction, cell survival, and neurite outgrowth [76, 113, 114]. Further, 3D porous chitosan scaffolds combined with NGF had a synergistic effect on the differentiation of neural stem cells and showed potential to regenerate damages in both CNS [115, 116] and PNS [117, 118].

Often, chitosan is used to enhance the biocompatibility of synthetic polymers with better mechanical characteristics. For example, chitosan was used to increase the biocompatibility of PVA in nanofibrous scaffolds, enhancing viability and proliferation of PC12 cells *in vitro* [26], and in PVA/SWCNTs structures, increasing the *in vitro* proliferation rate and integration of human derived brain cells U373 [119]. Aligned PCL/chitosan fibres supported PC12 cells adhesion and growth, enhancing neurite extension along the fibre orientation [120]. PLGA/chitosan scaffolds guided neuronal differentiation for peripheral nerve regeneration both *in vitro* and *in vivo* [121, 122].

In addition, chitosan can be chemically modified with ease, thanks to its ability to absorb cell-adhesive molecules, such as collagen, fibronectin, laminin, and genipin. These molecules react with the proteins on the surface of Schwann cells and support their attachment and proliferation, showing potential in directing peripheral nerve regeneration [123–125].

Chitosan micro/nano vehicles have also been successfully developed to deliver antitumor drugs, growth factors, and pharmaceutical medications to the CNS. For example, Skop et al. designed and optimised biocompatible chitosan microspheres for the delivery of neural stem cells and growth factors for CNS injuries [126] while Elnaggar et al. designed chitosan particles loaded with the drug piperine, reported to have neuroprotective potential against Alzheimer’s Disease, which successfully targeted specific areas of the brain [34]. Chitosan nanoparticles have also been developed for intranasal delivery of therapeutic agents to the brain [127, 128].

Chitosan has found recent application as a novel bioink for neural applications and 3D printing of neural constructs. Gu et al. developed a combination of extruded chitosan, alginate, and agarose to form a bioink seeded with front cortical human neural stem cells. Immediately after printing 25% of the seeded cells died, but the cells proliferation continued and, after three weeks, immunohistochemical analysis showed signs of mature neurons [129].

### Keratin

Keratin protein is a polypeptide composed of different amino acids with intermolecular bonding of the disulphide cysteine amino acid and inter and intra-molecular bonding of polar and non-polar acids. Keratin has demonstrated great potential as a biomaterial and it has a long history of applications in the biomedical field due to its high performance biological functionalities. Keratin’s ability to create suitable substrates and bioscaffolds is linked to its optimal biodegradability, biocompatibility, and non-immunogenicity. Keratin also facilitates good cell adhesion and proliferation through its biological characteristics and its versatile amino acid structure can be easily modified to suit a particular tissue [130].

Keratin was one of the first biomaterials to show promise for neural tissue engineering, due to its biological activities which facilitated the proliferation and infiltration of Schwann cells [131]. Keratin has found widespread application in the treatment of peripheral nerve injuries. In particular, keratin hydrogels can build biocompatible structures that facilitate neural cell adhesion and axonal ingrowth, while being reliably biodegradable. Multiple studies showed how keratin hydrogels promote the rapid regeneration of peripheral nerves *in vivo*, enhancing the activity, attachment, and proliferation of Schwann cells. Keratin hydrogels have the potential to be used clinically to improve conduits repair, producing long-term electrical and histological results equivalent to sensory nerve autograft. However, some of these older studies [132, 133] only considered small nerve gaps (4 mm).

Recent studies have tried to bridge a more significant nerve gap. For example, Lin et al. have developed a keratin hydrogel that, in combination with PCL nerve guides, allowed the group to bridge a 15mm sciatic nerve injury in a rat model, promoting Schwann cell and axon migration [134]. Hill et al. used keratin hydrogel scaffolds to bridge a considerable gap of 2cm in a rabbit model, and, although the keratin conduits were not as successful as nerve autografts, they induced a significant improvement in the overall recovery [135]. Pace et al. used a keratin nerve conduit luminal filler to bridge a 1cm nerve gap in primates, demonstrating the effectiveness of keratin as a biomaterial for nerve regeneration when confronted with a saline-treated control group [136].

Keratin can be easily electrospun in combination with other non-natural polymers with stronger mechanical characteristics, enhancing their biocompatibility with excellent results. Electrospun keratin fibres consistently showed good biocompatibility, cell attachment, proliferation, and viability. In neural tissue engineering, an electrospun PVA/keratin nanofibrous scaffold allowed glial cells adhesion, proliferation, and viability *in vitro*, confirming the optimal results seen in other tissue engineering fields [137].

### Silk

Silk is a fibrous structural protein produced by silkworms and spiders with unique properties suitable for a biomaterial. Silk shows great mechanical strength, excellent biocompatibility, minimal immunogenicity, limited bacterial adhesion, and controllable biodegradability [138]. Further, silk is a versatile material that has been used for the fabrication of biomimetic structures, such as films, hydrogels, scaffolds, nanofibres, and nanoparticles.

Kaplan’s intensive research on silk has uncovered novel applications for neural tissue engineering [30, 39, 139]. In particular, silk hydrogels are soft and sustainable biomaterials often used in neural tissue engineering due to their ability to maintain structural integrity more than other biomaterials, such as fibrin and collagen gels, while being able to elicit increased axonal bundling. Silk hydrogels have been successfully developed as functional scaffolds to support the differentiation of neurons for the regeneration of brain and nerve tissue [30, 139, 140]. Silk hydrogels can also be chemically modified with bioactive peptides, such as IKVAV, that increased cell viability and enhanced neural differentiation [141]. Further, silk hydrogels showed potential for 3D bioprinting of functional nerve tissue, characterised by high resolution, low feature size, reproducibility, and long term cell viability [142].

Silk fibroin showed good biocompatibility and absence of cytotoxic effects *in vitro*, and it can act as tissue engineered nerve guide for potential treatment of CNS injuries. For example, Benfenati et al. developed silk fibroin films that supported neurite outgrowth and preserved neuronal functions, such as the intracellular Ca^2+^ concentration response to a noxious stimulus [143]. Zhang et al. created a 3D silk fibroin scaffold with uniaxial channels that provided continuous contact guidance and regulated axonal elongation for the maturation of hippocampal neurons [144]. Recently, Gennari et al. developed a silk fibroin scaffold for *in situ* delivery of gamma-aminobutyric acid (GABA) and allopregnanolone (ALLO) showing strong Schwann cells attachment and neuronal survival [145].

Further, silk fibroin can be easily electrospun for nerve tissue engineering applications. For example, Tian et al. developed electrospun PLA/silk fibroin nanofibres embedded in NGF, that supported attachment and differentiation of PC12 cells [146]. Dinis et al. developed an electrospun silk-based nerve conduit with aligned and longitudinally oriented microchannels aimed at peripheral nerve regeneration. The nerve graft mechanical behaviors were comparable to those of rat sciatic nerves, showing a similar stress-strain behavior and tensile strength [39]. Xue et al. investigated the regenerative properties of an electrospun silk-fibroin scaffold in a long, 30mm, sciatic nerve lesion in dogs, showing results close to those achieved by autologous nerve graft [24]. Combinations of silk and electroconductive polymers also demonstrated potential for peripheral nerve regeneration. Das et al. fabricated an electrospun PANi/silk conduit that showed excellent biocompatible and electrophysiological parameters after 12 months of implantation in rats, exhibiting cellular recruitment and thick lamellar deposition of myelin over regenerating axons [40]. Silk/CNTs composite scaffolds have consistently shown excellent results in neural tissue engineering, promoting neural differentiation and serving as an efficient supporting matrix for the regeneration of nerve tissue [147, 148].

In addition, silk coating around brain-penetrating electrodes reduced glial scarring in the CNS, allowing the implantation of electrodes for electrophysiological recording and local stimulation *in vivo,* used for diagnostic and therapeutic purposes [149, 150].

Spider silk is less used in neural tissue engineering mainly due to difficulties in retrieving the material, but its application *in vitro* has been successful. For example, Roloff et al. used spider silk as a guidance conduit for human model neurons, inducing the formation of ganglion-like cell structures in four weeks [151], while Lewicka et al. developed a spider silk matrix that provided an optimal microenvironment for neural stem cells cultures [152].

## Synthetic polymers for neural tissue engineering

Synthetic polymers used for neural applications can be either biodegradable or non-biodegradable. Polyesters of lactic and glycolic acid, PLA, PGA, and their co-polymer PLGA, are considered biodegradable, like hydrogels based on polyethylene glycol, PEG, whereas biomaterials containing methacrylate are usually non-biodegradable. Initially, neural scaffolds were made of the same materials used for surgical repairs of peripheral nerves and skin grafting [49]. However, due to advances in biomaterials chemistry and technology, new matrices have been created that better suit the neural environment [153]. Nowadays neural scaffolds are principally highly aqueous hydrogels, soft polymers that share various similarities and properties with the nerve tissue, and present a strong versatility, which allows their chemistry and architecture to be adjusted according to a specific need [109, 135, 139]. Functionalization of synthetic polymers through surface modification techniques and inclusion of neurotrophic factors expanded the use of synthetic scaffolds to drug delivery and gene delivery vehicles to the CNS [154–156].

The use of synthetic or non-natural polymers in neural tissue engineering is advantageous because of their mechanical strength and flexibility combined with ease of modification and tailorability, as their structural properties can be modified in many ways, including blending and copolymerization. Synthetic polymers are also compatible with numerous fabrication techniques, such as wet-spinning, freeze-drying, and electrospinning. However, there are inherent problems with the use of synthetic polymers. Despite synthetic polymers being mainly non-toxic, there are still concerns regarding toxic residual monomers from incomplete polymerisation as well as degradation products and plasticisers. Therefore, synthetic polymers require intensive and comprehensive testing prior to translation into the clinic. The main synthetic polymers used in neural tissue engineering have been summarized in Table 2 along with their applications.

**Table 2:** The main synthetic polymers used in neural tissue engineering, biocompatibility *in vitro/in vivo*, and examples of their applications

Synthetic Polymer	Biocompatibility *in vitro*	Biocompatibility *in vivo*	Application	References
Preclinical Studies				
PLA	Rat SCs		Nanofibrous conduit	[159]
	NSCs		Nanofibrous conduit	[162, 163]
PLGA	U87		Microparticles	[36]
		Dogs	Nerve conduit	[122]
	DRGs	Rats	Scaffold	[164]
		Rats	Microparticles	[166]
PEG	NPCs		Hydrogel	[169–171]
		Rats	Hydrogel	[171]
		Rats	Intravenous administration	[172, 173]
	Guinea pig spinal cord injury model		pure PEG	[174]
		Rats	Scaffold	[175]
pHEMA		Rats	Hydrophilic sponge	[176]
	PC12		Hydrogel	[179]

*PLA*: polylactic acid; *PLGA*: poly(lactic-co-glycolic acid); *PEG*: polyethylene glycol; *pHEMA*: poly(2-hydroxyethylmethacrylate); *SCs*: stem cells; *NSCs*: neural stem cells; *DRGs*: dorsal root ganglia; *NPCs*: neural progenitor cells.

### Synthetic polymers

Poly (α-hydroxy acid) polymers such as poly(lactic acid), (PLA), poly(glycolic acid), (PGA), and their copolymer poly(lactic-co-glycolic acid), (PLGA), have been used as biomaterials for a number of different biomedical applications focusing on neural tissue engineering. PLA and PGA are thermoplastic polymers characterized by polyesters links of, respectively, lactic or glycolic acid. Both PLA and PGA can be absorbed or hydrolyzed *in vivo,* and they are biodegradable. PLA and PGA were the first biopolymers trialed for regenerate studies using nervous tissue as they had been previously used as an absorbable suture material [157] and grafting material for wound healing [158].

PLA has been successfully used to design and construct scaffolds that provide support to Schwann cells, allowing elongation of axons, and to promote vascular growth [159]. However, PLA scaffolds have been found to be dimensionally or structurally unstable, often shattering and crumpling. Equally, PGA-based nanoconduits have excellent mechanical properties that favor their use in clinical settings, but it was demonstrated that they progressively lose their strength after 1-2 months upon implantation [160]. Due to their instable nature, in most cases PGA-based nanoconduits are limited to bridge a small nerve gap [161]. Based on these data, researches prefer to use PLA or PGA copolymers as they are more mechanically reliable.

PLA based multichannel conduits with a nanofibrous microstructure have been used to promote the differentiation of neural stem cells in mature neurons *in vitro* [162]. Despite the improved mechanical characteristics, the nanofibrous microstructure was still observed to degrade too fast and it had to be stabilized using a natural polymer, in particular through the use of a gelatin wrap [163].

PLGA has been used extensively in neural tissue engineering because its characteristics, including permeability, swelling, deformation, and degradation rate, can be controlled by altering the ratio of PLA:PGA to suit specific applications, especially drug delivery microparticles and conduits for nerve regeneration. For instance, multichannel PLGA scaffolds seeded with Schwann cells have been shown to have a synergistic effects on neural regeneration, albeit further studies are needed to aid functional recovery [122, 164]. Further, PLGA seems to be extremely effective in transporting therapeutic agents across the BBB. The inherent difficulties that the BBB represents as a protective layer has forced researchers to engineer multiple different approaches that attempt to deliver substantial quantities of drugs to specific parts of the brain. PLGA microspheres have shown potential to overcome this crucial issue and cross the BBB to deliver anti-tumor drugs [36, 165] and glial derived neurotrophic factor (GDNF) for the treatment of neurodegenerative diseases such as Parkinson’s Disease [166].

Hydrogels are three-dimensional cross-linked hydrophilic polymer networks capable of swelling and de-swelling reversibly in water and can retain large volumes of liquid in a swollen state [167]. They can be designed to have controllable responses and shrink or expand according to the environment they are in [167]. In neural tissue engineering, the two most common polymers used to create synthetic hydrogels are polyethylene glycol (PEG) and poly (2-hydroxyethyl methacrylate); (pHEMA).

PEG is a biodegradable synthetic polymer of ethylene oxide (EO) units. PEG is highly biocompatible and well suited for use in hydrogels due to its hydrophilic properties, crucial for nutrient and waste transport, and is also biochemically inert. In addition, PEG is non-immunogenic and resistant to protein absorption. However, unlike natural polymers used in hydrogels, PEG is not bioactive, hence it is often used in combination with other polymers [168].

PEG hydrogels have been extensively used for neural tissue engineering. Neuronal cell growth on PEG platforms improve neural cell survival, proliferation, and differentiation, showing great potential for the treatment of CNS injuries [169–171]. Hydrogels can be designed and constructed in different shapes (2D or 3D), but the properties and attributes of the design seem to influence neuronal cells behavior more so in 3D cultures rather than 2D cultures. Lampe et al. showed that in PEG 2D cultures, hydrogel properties did not affect metabolic or apoptotic activity, but did impact cell proliferation and increased glial cell reactivity. Conversely, in PEG 3D cultures that closely matched the stiffness of the native brain, cells had increased metabolic activity and proliferation and lower rates of apoptosis [169].

The versatile and non-toxic nature of PEG meant it could be trialed preclinically and administered intravenously following head injury. After severe traumatic brain injury, intravenous administration of PEG dampened cerebral cell loss and slowed the degeneration of injured axons to the point that PEG-treated brains closely resembled those of uninjured animals [172, 173]. Further, PEG has shown promise following spinal cord injury, significantly accelerating and enhancing the membrane resealing process and restoring mechanical integrity following compression [174]. Liu et al. also showed enhanced cell growth and migration along with improved functional recovery in rats after transection of the spinal cord using an electrospun PLGA/PEG scaffold [175].

pHEMA, is a polymer that forms hydrogels in water due to its extremely hydrophilic nature, is biocompatible, and it can polymerise at low temperatures, (between -20°C and +10°C), which allows immobilisation of proteins into hydrogels.

**Fig. 4:**
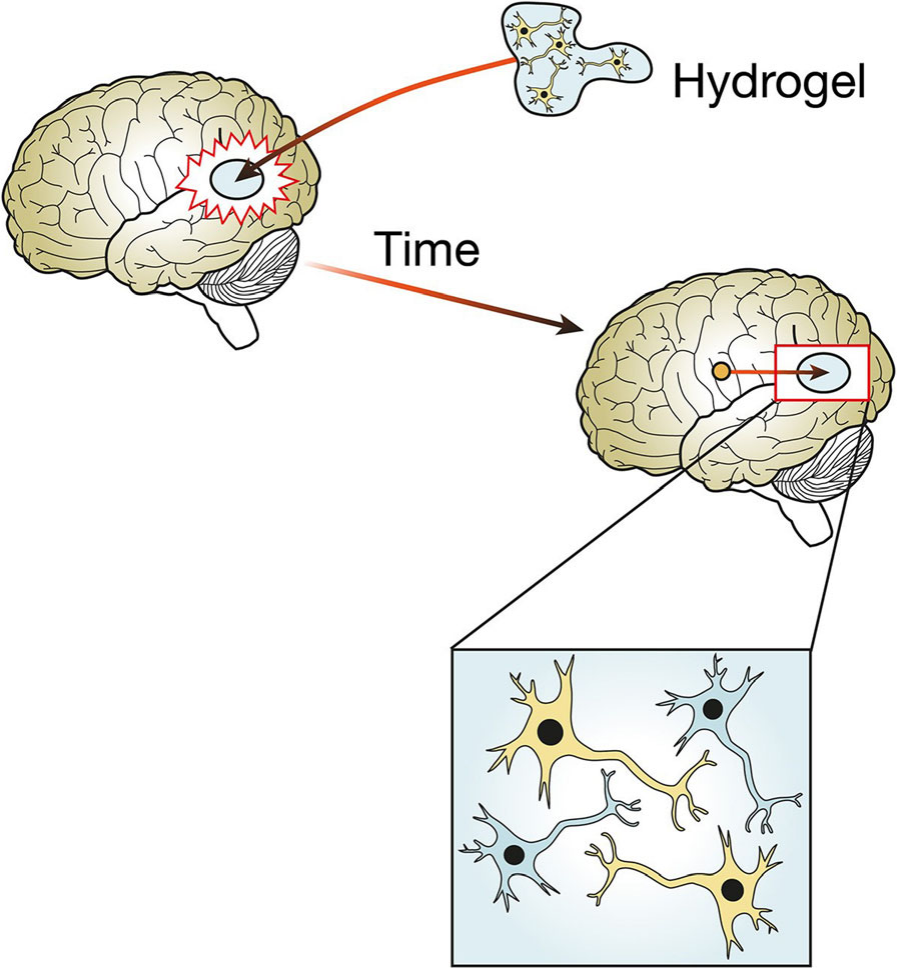
Polymer hydrogel supports the regeneration of the brain tissue. Stroke causes reactive astrocytes to inhibit the regeneration of the brain tissue. A polymeric hydrogel seeded with neural cells is surgically implanted into the cavity caused by the stroke. With time, the reactive astrocytes are mitigated and the host’s neurons can communicate with the cells seeded inside the hydrogel, reforming neural connections and restoring the original functions of the brain tissue

Initially, pHEMA was developed as a hydrophilic sponge, wherein porous hydrophilic sponges provide a stable three-dimensional scaffold capable of supporting and promoting axonal regeneration in rats [176]. Subsequently, Shoichet’s group in Canada has development multiple pHEMA hydrogels capable of guiding neurite outgrowth. Figure 4 represent a simple schematic of an implanted hydrogel establishing new functional reconnections between seeded cells and the host’s neurons. By controlling the formulation and surface chemistry, pHEMA hydrogels can be tailor made to suit a particular application in either the CNS or the PNS. For example, pHEMA hydrogel tubes have been manufactured with similar mechanical properties to those of the spinal cord, with a reported elastic modulus ranging between 200 and 600 kPa [177]. Further, some pHEMA hydrogels were more suitable for peripheral nerve regeneration [178], and others have been developed with a photochemically bound nerve growth factor that promoted neurite outgrowth on 3D surfaces *in vitro* [179].

One of the main advantages of the use of hydrogels, both PEG and pHEMA, in neural tissue engineering is the variety of characteristics that can be achieved. They can be applied as nerve guidance conduits, intravenous inhibitors of cell death, and as 3D structures that support the formation of nervous tissue. The array of complex architectures that can be accomplished guarantees the ability to tailor each polymer to a specific application, mimicking the environment of the host tissue.

### Electrically conductive polymers

The nervous system operates through a delicate network of electrical signaling in order for neurons to communicate with other cell types. Ideally, neural scaffolds should possess electrical properties to enhance proliferation and migration of neuronal cells [180]. Therefore, an electrically conductive polymer could ideally mimic the neural tissue and facilitate the reconstruction of neural connections.

Electrically conductive polymers have loosely held electrons along their backbones. In order to purposefully manipulate the electrical properties of conductive materials, they have to undergo a process called doping. The process of doping for conductive polymers usually include adding chemicals reactants to oxidize or reduce the systems so that electrons are pushed into the conductive orbital within the potentially conducting system. Electroconductive polymers have attracted attention in neural tissue engineering because of their tunable properties, including good stability, electrical conductivity, and ability to encapsulate and release molecules. Further, their electrical, chemical, and physical properties can be modified to suit a specific application. However, a critical issue in the use of conductive polymers as biomaterials for neural tissue engineering is suboptimal biocompatibility linked to their inability to degrade *in vivo,* which could induce chronic inflammation, and immunogenic reactions, requiring additional surgeries and treatments [181]. To overcome this issue, conductive polymers have been systematically blended with other biodegradable polymers, both synthetic and natural in origin, combining crucial electroconductive properties with more biologically favorable biomaterials. Table 3. summarizes the main electrically conductive polymers used in neural tissue engineering and their applications.

**Table 3:** The main electroconductive polymers used in neural tissue engineering, biocompatibility *in vitro/in vivo*, and examples of their applications

Electroconductive Polymer	Biocompatibility *in vitro*	Biocompatibility *in vivo*	Application	References
Preclinical Studies				
PPy	PC12		Electrospun nanofibers	[19, 182]
	PC12	Rats	Films	[20]
	PC12	Rats	Nerve conduit	[183, 184]
	hNSCs	Rats	Hydrogel	185, 186]
	PC12		Electrodes	[187]
	Auditory neurons from Albino-Wistar rats		Electrodes	[188]
	Primary murine cerebellar glial culture		Vehicle for drug delivery	[190]
	PC12		Vehicle for drug delivery	[154]
		Rats	Vehicle for drug delivery	[191]
PANi	PC12		Electrospun nanofibres	[193]
		Rats	Nerve conduit	[38, 194]
	NSCs		Hydrogel	[195]
	PC12		Hydrogel	[31]
		Rats	Electronic patch	[196]
PEDOT		Rats	Electrodes	[197, 198]
		Mice	Electrodes	[46]
	Neurons from E18 Sprague- Dawley cortices		Electrodes	[199]
		Rats	Electrodes	[200, 201]
	ReNcell VM		Substrate	[202]
	P19		Substrate	[203]
InP	Primary cell cultures from hippocampal regions of rats		Nanowire scaffolds	[204]

*PPy*: polypyrrole; *PANi*: polyaniline; *PEDOT*: Poly (3,4-ethylenedioxythiopene); *InP*: indium phosphide; *hNSCs*: human neural stem cells; *NSCs*: neural stem cells.

Polypyrrole (PPy) is an organic polymer formed by the polymerization of pyrrole monomer and it is one of the most commonly used conductive polymers in neural tissue engineering. PPy is mainly used in combination with other biodegradable non-natural polymers such as PLA, PLGA, and PCL, in order to enhance its biocompatibility. For example, PPy-coated PLGA electrospun nanofibres combined the effect of electrical stimulation and topographical guidance that resulted in increased neurite growth [182]. Further, PPy-PLA fibres have been shown to enhance neurite adhesion, alignment, and elongation [19], whilst PPy-PCL films has been shown to support cell proliferation and enhance neurite outgrowth through electrical stimulation both *in vitro* and *in vivo* [20]. Finally, a PPy-PDLLA nerve conduit has been used to repair sciatic nerve injury in rats, performing to the degree of the clinical gold standard in terms of functional recovery [183, 184]. PPy, combined with natural polymers, such as hyaluronic acid, has also been used to construct three dimensional electroconductive hydrogels aimed at improving recovery from traumatic brain injuries and stroke [185, 186].

One of the most interesting applications of PPy is its use as a new electrode material for long-term chronically implantable neuroprosthetics devices [187, 188]. Recently, PPy immersion in plasma has been shown to limit adverse immune reactions and favor direct tissue integration. Kondyurin et al. used this technique to create a biologically active electro-stimulating neural interface [189]. PPy has also found applications as an electrically controlled vehicle for localized drug delivery to the CNS [154, 190, 191].

Polyaniline (PANi) is another useful conductive polymer that has many attractive properties, including high conductivity, easy synthesis, low cost, and easy availability [192]. Similar to PPy, a critical issue for the use of PANi in neural tissue engineering is its suboptimal biocompatibility. Therefore, PANi is often used in combination with more suitable biodegradable polymers, to mitigate inflammations or immunogenic reactions. For example, PANi/PLA-PCL electroactive electrospun fibres enhanced the NGF-induced neurite outgrowth of PC12 cells and showed great potential for nerve regeneration as an effective graft material [193]. Further, a research group in China has also successfully combined PANi with the natural biopolymer cellulose aiming at peripheral nerve regeneration, leading to possible clinical intervention for nerve injuries [38, 194].

Like PPy, PANi has been designed as a hydrogel material for neural tissue engineering. In particular, PANi hydrogels have been developed for peripheral nerve regeneration and as substrates for neural stem cells differentiation [31, 195]. In addition, PANi has recently showed promise as a biosensing electronic patch that could be integrated in electroresponsive tissues for recording and therapeutic stimulation, opening a new range of possible research trends for this material in neural tissue engineering [196].

Poly (3,4-ethylenedioxythiopene), (PEDOT), is an interesting electroconductive polymer characterised by optical transparency in its conductive state, high stability, and low redox potential. PEDOT has found numerous applications in neural tissue engineering, especially as a material for microelectrodes aimed at neural electrical stimulation and recording [46, 197, 198]. In particular, Cui’s research group has developed numerous PEDOT-based electrodes for stable neural recording and therapeutic stimulation [199–201]. The use of PEDOT in neural tissue engineering has recently expanded to include neural stem cell differentiation through electrical stimulation, leading to longer neurite outgrowth and longer neurons [202, 203].

Furthermore, a recent study has shown that indium phosphide (InP) nanowire scaffolds influence neuronal and cell morphology, circuit formation, and function [204]. InP is a direct band semiconductor which is usually utilized for superior optoelectronic interfaces, and due to its conductive abilities, it is an effective physical cue for guided growth of neurites. This is the first work that showcases how neural cells can grow on InP-based optoelectronic substrates, proving its biocompatibility, and opening a new scenario for therapeutic approaches using electroconductive polymers [204].

### Carbon-based nanomaterials

Carbon-based nanomaterials present exclusive electrical, mechanical, and biological characteristics, which make them particularly useful for tissue engineering. In particular, neural tissue engineering mainly utilizes graphene and carbon nanotubes because of their inherent properties of conductivity, flexibility, and biocompatibility. However, there is a substantial gap in our knowledge in terms of understanding the biological interactions of carbon-based nanomaterials *in vivo*. Therefore, further investigation is needed in order to explore interactions of carbon-based nanomaterials with singular cellular components and components of the immune system, as well as mapping the ultimate cycle of the materials *in vivo*, including accumulation, degradation, and/or excretion. Table 4 summarizes the uses of graphene and carbon nanotubes in neural tissue engineering.

**Table 4:** The main carbon-based nanomaterials used in neural tissue engineering, biocompatibility *in vitro/in vivo*, and examples of their applications

Electroconductive Polymer	Biocompatibility *in vitro*	Biocompatibility *in vivo*	Application	References
Preclinical Studies				
Graphene	PC12		Sheets	[205]
	BV2 cells		Foam	[206]
	NSCs		Substrate	[35]
	NSCs		Rolled foam	[25, 207]
	hNSCs		Nanogrids	[208–211]
		Rats and Mice	Electrode	[212]
CNTs	NG108-15		SWCNTs	[213]
	Hippocampal neuronal cultures from Sprague-Dawley rats		SWCNTs	[215]
	Astrocytic cultures from C57BL/6 mice pups		SWCNTs	[216]
	NSCs		SWCNTs	[217]
	NSCs		MWCNTs	[218]
		Mice	MWCNTs	[219]
	PC12		MWCNTs	[220]
	PC12		Electrodes	[187]
		Rats	Electrodes	[221]

*hNSCs*: human neural stem cells; *NCSs*: neural stem cells; *CNTs*: carbon nanotubes; *SWCNTs*: single-walled carbon nanotubes; *MWCNTs*: multi-walled carbon nanotubes.

Graphene is an allotrope of carbon consisting of a single layer of carbon atoms arranged in a 2-dimensional hexagonal lattice. It efficiently conducts heat and electricity, it is nearly transparent, bactericidal, and antiviral, and it is highly biocompatible with low cell toxicity. However, it should be noted that depending on whether graphene is used in 2D or 3D, matrix cellular toxicity has been reported [205, 206], with 3D cultures being better for neural cell growth and proliferation. In addition, 3D graphene substrates are also able to encapsulate different nanoparticles, such as gold, increasing neuronal differentiation and guiding axonal alignment [35].

Graphene has been used for neural tissue engineering applications in numerous forms, particularly foams and graphene nanogrids. Rolled graphene foams have been developed as electrically conductive 3D scaffolds that stimulate and accelerate differentiation and proliferation of human neural stem cells [25, 207]. Akhvan’s research showed that graphene nanogrids also increase the neural cell to glial cell ratio thanks to biocompatible stimulation techniques, including electrical, pulsed laser, flash photo, and near infra-red (NIR) laser stimulation [208–211]. Graphene has also found interesting applications as a material for neural probes, enhancing the quality of the neural-device interface [212].

Carbon nanotubes (CNTs) are allotropes of carbon with a cylindrical structure characterized by extraordinary thermal conductivity and optimal mechanical and electrical properties. CNTs are excellent candidates for neural tissue engineering due to their biocompatible, conductive, and non-biodegradable nature. Primarily, CNTs function as implants where long-term cues for neurite outgrowth are necessary, such as regeneration after spinal cord injury or brain injury [213]. Both single walled carbon nanotubes (SWCNTs) and multi walled carbon nanotubes (MWCNTs) have been used in neural tissue engineering. SWCNTs work as substrates that modulate and stimulate neural cells through the variation of conductance, for example using lateral currents for the purpose of healing neurological and brain related injuries [214]. SWCNTs substrates also promoted neurite outgrowth *in vitro* [215–217]. Conversely, MWCNTs, due to greater stability, have been applied to novel technologies such as 3D printing of scaffolds for peripheral nerve regeneration [218], and developed as neural guidance conduits and as targeted drug delivery vehicles to the CNS [219, 220].

Finally, CNTs-based electrodes have been developed for chronically implantable neural interfaces and recording of electrogenic cells [187, 221].

## Conclusion and future perspectives

This review highlighted the most prominent polymeric biomaterials that demonstrated potential for neural tissue engineering. Given the degree of morbidity and lifelong impairments caused by injuries to the nervous system, both CNS and PNS, its functional restoration is critical. Current therapeutic approaches for damages to CNS and PNS are insufficient to restore the functions of the nervous system and new tissue engineering applications are needed. This review investigated the main polymers, both synthetic and natural, used in neural tissue engineering, focusing on their biocompatibility both *in vitro* and *in vivo*, key applications, and advantages/disadvantages between different kinds of polymers. Polymeric applications for neural tissue engineering continue to evolve rapidly, focusing in particular on biocompatible electroconductive materials, such as PPy and PANi. The use of growth factors, neural progenitor cells, and neural stem cells combined with biopolymeric structures has improved the restoration capacities of polymeric biomaterials as well as their ability to target specific areas of the CNS and deliver therapeutic agents. Further advances in neural tissue engineering need to focus on innovative combinations of biopolymers and proteins, such as silk or keratin, which have shown great potential for new therapeutic and regenerative applications. Further, novel fabrication techniques are being explored and electrospinning has clearly emerged as an optimal candidate for neural tissue engineering, due to its ability to control the orientation of electrospun nanofibres, following the preferred directionality of neurite outgrowth. It is auspicious that continuous scientific research on promising natural polymers, regenerative strategies, and fabrication techniques will maximise the ability to functionally regenerate nervous tissues and restore electrochemical connections, unburdening society from the disastrous consequences of damages to CNS and PNS.

## Abbreviations

*ALLO*: Allopregnanolone
*BBB*: Blood Brain Barrier
*CNS*: Central Nervous System
*CNTs*: Carbon Nanotubes
*ECM*: Extracellular Matrix
*ELPs*: Elastin-like polypeptides
*GABA*: Gamma-aminobutyric acid
*GDNF*: Glial Derived Neurotrophic factor
*HA*: Hyaluronic Acid
*hNSCs*: Human Neural Stem Cells
*InP*: Indium Phospide
*NGF*: Neural Growth Factor
*PANi*: Polyaniline
*PCL*: Polycaprolactone
*PEDOT*: Poly(3,4-ethylenedioxythiopene)
*PEG*: Polyethylene glycol
*PGA*: Poly-glycolic acid
*pHEMA*: Poly(2-hydroxyethylmethacrylate)
*PLA*: Poly-lactic acid
*PLGA*: Poly-lactic-co-glycolic Acid
*PNS*: Peripheral Nervous System
*PPy*: Polypirrole
